# Revisiting Fluoride in the Twenty-First Century: Safety and Efficacy Considerations

**DOI:** 10.3389/froh.2022.873157

**Published:** 2022-07-04

**Authors:** Steve Duffin, Marcus Duffin, Martin Grootveld

**Affiliations:** ^1^Shoreview Dental LLC, Keizer, OR, United States; ^2^NoDK LLC, Wilsonville, OR, United States; ^3^Oral Health Outreach LLC, Wilsonville, OR, United States; ^4^Leicester School of Pharmacy, De Montfort University, Leicester, United Kingdom

**Keywords:** fluoride, silver, silver diammine fluoride, monofluorophosphate, stannous fluoride, health and safety, toxicity, dental caries

## Abstract

Over 100 years of scientific literature is available which describes the long relationship between dentistry and the many possible applications of fluoride anion (F^−^) as successful therapeutic strategies. To date, systemic introduction of fluoride *via* water, milk and salt fluoridation, and fluoride-containing tablets, has been employed. Post-eruption topical fluoride products have also been introduced, such as fluoridated toothpaste, along with fluoride-containing rinses and varnishes. Importantly, a recent addition to the available armamentarium of fluoride therapeutics now exists in the form of metal ion-fluorido adducts, most especially silver(I)-diammine fluoride (SDF). In view of its high level of therapeutic success, very recently this agent was added to the World Health Authority's (WHO's) list of essential medicines available for the treatment and prevention of tooth decay. Overall, this current state of affairs merits a major review of all these fluoride-containing therapeutic compounds, together with their risks and benefits, both individually and collectively. In this study, a simple graphical tool has been developed for the rapid “on-site” evaluation of fluoride intake with respect to a range of oral healthcare products and body mass index is presented as a gauge of safety for the risk of fluoride toxicity in individual patients. This exposition commences with (a) an account of the history and value of fluoride therapeutics in clinical dentistry, including applications of monofluorophosphate and stannous fluoride; (b) an evaluation of the toxicological activities of fluoride, together with a summary of any reports, albeit very rare ones, arising from its clinically-driven overuse; (c) a history of the development, molecular structure, mechanisms of action, and therapeutic applications of SDF, including a summary of any possible toxic activities and effects arising from silver(I) ion rather than fluoride itself; and (d) the establishment of a working relationship between fluoride exposure and toxicity, with special reference to the instigation of newly-developed tabular/graphical reference guidelines for use by dental clinicians who employ fluoride-rich products in their practices. Particular attention is given to the oral care and treatment options of pediatric patients. In conclusion, applications of this unique monitoring tool may serve as a valuable toxicity guide for dental practitioners.

## Introduction: Fluoride Use in Dentistry and Oral Health

**Figure 1:**
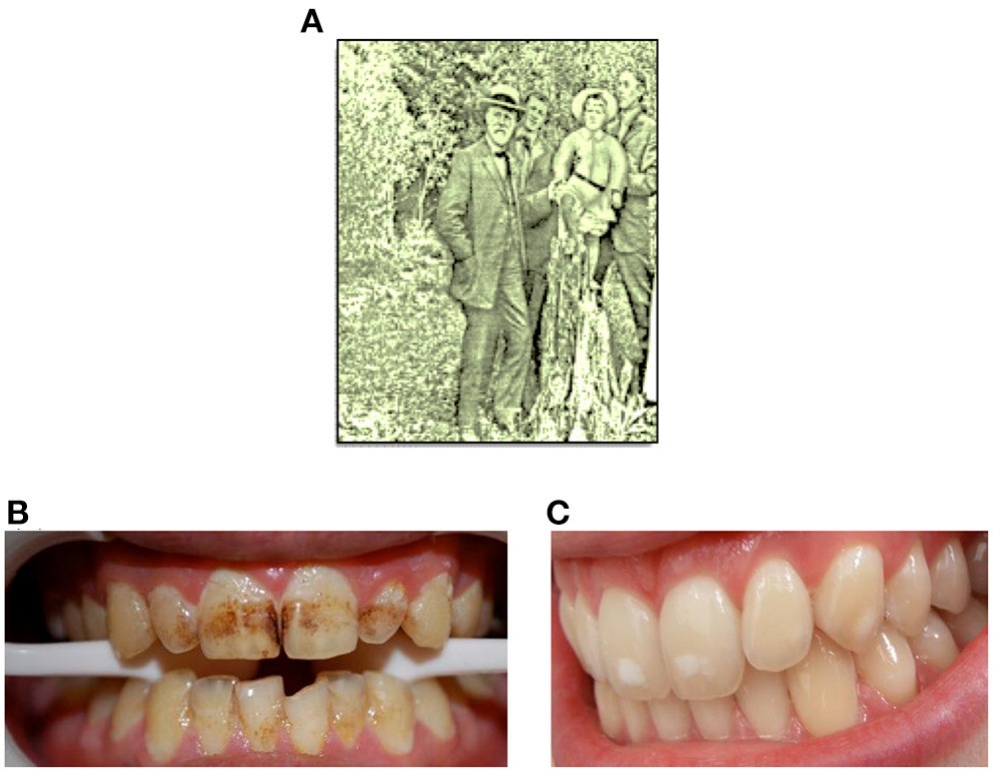
**(A)** Dr. Black in Colorado Springs, 1909. **(B,C)** Typical photographic representations of “Colarado brown stain” and mild mottled enamel, respectively.

In 1901, the dentist Fredrick McKay working in Colorado Springs, Colorado noticed a condition in many of his patients which he referred to as “Colorado Brown Stain” (Figure 1B) [1]. In order to attempt to understand this phenomenon, Dr. McKay requested the assistance of Dr. G. V. Black from Northwestern University (Figure 1A) to facilitate the development of an explanation for the possible cause of this clinical finding. Dr. Black identified the enamel lesions as hypomineralized areas, which he characterized as “mottled enamel” (Figure 1C), and which he assumed to be at a higher risk of further demineralization [2, 3].

However, one surprising and counter-intuitive discovery made by McKay and Black was that patients with the so-called “Colorado brown stain,” or severely mottled enamel, had a diminished rate of caries development than that of other patient groups. McKay speculated that this phenomenon might arise from some chemical agent present in local drinking water [4], and in the 1930's, systematic animal experiments and human epidemiological studies demonstrated a cause-and-effect relationship between fluoride (F^−^) levels present in drinking water and mottled enamel [3].

A full outline of the history of fluoride use in dentistry and oral health is beyond the scope of this manuscript, but particularly notable are the works of H. Trendley Dean, who continued to explore relationships between water fluoride levels, mottled enamel, and tooth decay. These efforts led to the proposal that an optimal level of fluoride in drinking water may minimize the negative cosmetic effects of mottled enamel, and also maximize the protective benefits offered against dental caries. This optimized fluoride level was proposed to be 1 ppm [5]. Moreover, the performance of a carefully controlled clinical trial in 1944 (based in Michigan, USA), along with additional studies conducted both in the USA and globally [1], confirmed highly valuable and resoundingly significant reductions in caries rate when fluoride was added to drinking water. In 2001, the *United States Centers for Disease Control and Prevention* identified water fluoridation as one of the 10 most important public health interventions of the 20th century [6]. A detailed account of the history of the substantial public health benefits offered by fluoride in dentistry is provided in Ref. [7].

In the current study, the authors report the development of a relatively simple graphical tool for the rapid evaluation of patient fluoride intake with respect to the composition of a series of oral healthcare products, and recipient body mass index. This tool is readily employable for the purpose of monitoring the safety and potential deleterious health risks of such fluoride-containing products, particularly toward children, at patient points-of-contact. Indeed, it is proposed that its routine clinical application will serve to provide valuable toxicological guidelines for both clinical dentists and oral healthcare specialists alike, and which may be viewed and interpreted rapidly. In this context, a full consideration of working relationships between fluoride exposure and toxicity will serve as a valuable health and safety benefit to dental practice staff, including those who regularly employ 50% (w/v) silver nitrate or 38% (w/v) SDF aqueous solution therapies, both with and without the subsequent application of a 5% (w/v) fluoride varnish product. Moreover, the clinical implications for these methodological developments are discussed, along with future recommendations for fluoride therapeutics in oral health.

Uniquely for a Methods report with a predominantly clinical readership, the authors also provide valuable information regarding the precise molecular structures, the fluoride and metal ion [silver(I) and tin(II)] speciation status, potential mechanisms of action, and health and safety information for all fluoride-containing adducts considered, in addition to those of fluoride anion itself.

### Fluoride Anion (F^–^) as in Sodium Fluoride (Na^+^/F^–^)

Today, the use of fluoride as an additive to water supplies and by way of topical application products are common. There are two major effects of these fluoride products. The first involves the development of tooth enamel during the pre-eruption stage. When fluoride is available systemically during the maturation of enamel hydroxyapatite crystals, fluoride becomes incorporated into enamel prisms, forming a fluorapatite compound which is more resistant to acid dissolution. Secondly, an additional pathway involves the topical application of fluoride to erupted teeth. A normal cyclical process of enamel demineralization and remineralization occurs in erupted teeth, as pH levels fall and rise, respectively, in view of biofilm activity. If topical fluoride is available during the remineralization phase, fluorapatite is formed, and this, in turn, will create an acid-resistant surface. Currently, fluoridated toothpaste has become the major source of topical fluoride throughout the world [6]. The principal mechanisms of reduced caries rates in many populations around the world have been largely attributed to the application of topical fluoride *via* oral healthcare products such as these toothpastes [6].

A series of Cochrane reviews providing an overview of the evidence available on the abilities of fluoride therapies to prevent dental caries was reported by Marinho in 2014 [8]. Major findings from the reviews considered were that for topically-applied fluoride treatments, there were clear decreases in caries increment in permanent and primary dentitions for all forms of therapies and fluoride varnishes alone, respectively; an arrest of dental caries with topically-administered fluoride products was also revealed, this effect being independent of water fluoridation exposure level, or of other routes of fluoride delivery (their caries preventative actions was found to be enhanced in cases with elevated initial caries population degrees, notably when higher fluoride doses are applied, or when involving the supervision of children's employment of fluoride-containing toothpastes and oral rinses). Moreover, clear protective effects against dental caries and its prevalence in both children and adolescents were offered by the use of fluoride-containing toothpaste products—such products represent the commonest form of fluoride intake globally, and such effects were observed as much as those with the use of other topically-applied fluoride formulations such as oral rinses, gels and varnishes. Additionally, evidence available revealed that the use of a fluoride toothpaste, together with another class of topically-applied fluoride treatment, give rise to additive diminutions in dental caries when compared to those receiving only fluoride-containing toothpaste. Clear enhancements in preventative effects against dental caries were observed with increasing fluoride toothpaste contents when this level is ≥1,000 ppm (the actions of such products containing lower fluoride contents were unclear, however). Nevertheless, there remains some marginal evidence that commencing the application of fluoride toothpaste in children of ≤ 12 months of age may be linked to an elevated risk of fluorosis.

Reviews which considered alternative fluoride interventions and comparisons, and published in The Cochrane Library, were also evaluated in this Cochran review [8]. These included studies of the caries- preventative influences of fluoride supplements, slowly-releasing fluoride devices and fluoride milk formulas, along with sealants and fluoride varnishes.

Although fluoride-liberating monofluorophosphate (MFP) and stannous fluoride (SnF_2_) therapies are no longer very widely used in dentistry and dentifrices, we have nevertheless considered their cariostatic activities and mechanisms of action, along with their molecular structures and biological chemistry in Sections Monofluorophosphate (MFP) and Stannous Fluoride (tin(II)-fluoride, SnF_2_), respectively. Silver(I)-diammine fluoride (SDF) is covered in Section Silver(I)/Fluoride Ion-Containing Products and Their History: Silver(I)-Diammine Fluoride (SDF).

### Monofluorophosphate (MFP)

MFP, as its sodium salt, has previously found a considerable level of application in oral health products, usually toothpastes, in view of its now well-known cariostatic and microbicidal effects. Its molecular structure consists of tetrahedral [PO_3_F]^−^ structural units with an intact P-F bond, which is subject to hydrolysis through the actions of phosphatase enzymes *in vivo*, a process liberating free fluoride anion and inorganic orthophosphate. Investigations performed in an animal model system demonstrated that although there was no such hydrolysis in the stomach, this process occurred very rapidly in both the small intestine and the liver, but more slowly in blood [9]. In both rats and humans, no evidence for direct absorption of [PO_3_F]^−^ anion into blood circulation was obtained [9]. Hence, these observations support the low acute toxicity found for MFP, and also the lack of gastric irritation associated with its use.

In 1993, Holloway and Worthington [10] conducted a critical review of a meta-analysis to establish the relative therapeutic effectiveness of sodium MFP when evaluated against sodium fluoride. In addition to revealing some important study flaws, this investigation found that two and three studies favored sodium fluoride and sodium MFP, respectively, whereas no fewer than five of them should not have been incorporated into a meta-analysis process. However, the only two scientifically-conceived and performed investigations did not conclude with any advantages of either agent over the other.

### Stannous Fluoride (Tin(II)-Fluoride, SnF_2_)

The chemical bonding in stannous fluoride (SnF_2_), a complex with a tin(II) (Sn(II)) metal ion coordination center and fluoride ligands, has a quite a strong covalent character. This agent has been demonstrated to successfully control and avert both dental caries and gingivitis *via* its ability to facilitate enamel mineralization and alleviate inflammation and bleeding of the gingiva. It also potentially exerts a rather broad-spectrum microbicidal effect, and also has the capacity to significantly modify the microbial contents of dental biofilms. Its mechanism of action involves the deposition of a stable acid-resistant tooth surface coating, which comprises calcium fluoride generated *via* the actions of SnF_2_ on apatite, and its transformation to fluorapatite, processes involving the exchange of F^−^ ‘ligands' from Sn(II) to Ca^2+^. Both the Sn(II) center and F^−^ ligand moieties play roles in the development of anti-erosive properties, possibly by intensifying the degree of cross-linking between salivary proteins of the absorbent layer, for example mucins [11]. This process gives rise to a layer which is more resistant against erosive attack, and it is conceivable that Sn(II) ions may form inter-protein metal ion-centered bridges through coordination to oxygen- and/or nitrogen-donor amino acid residue complexants in these biopolymers. Sn(II) has a preference for oxygen-donor atoms in ligands available *in vivo*, although the cumulative stability constants for its fluoride complexes are indeed quite high [12]. Notably, the value and protective effects offered by SnF_2_ appear to be associated with the uptake of Sn(II) metal ion species by mineralized dentine containing a largely conserved organic component. Indeed, its ability to suppress erosion is critically dependent on the availability of a demineralized organic dentine environment [12].

Recently, Alsina and Gaillard [13] investigated the identities and structures of tin(II)-fluoride complexes in aqueous solutions *via* a combination of thermodynamic modeling, X-ray absorption spectroscopy, and quantum mechanical computations. Spectroscopic measurements confirmed the presence of three tin(II)-fluoride complexes in this medium (specifically [SnF]^+^, [SnF_2_] and [SnF_3_]^−^). Interestingly, in addition to the fluoride ligands, the [SnF_3_]^−^ complex also contained weakly coordinated water, which was displaced by glycerol added to the solvent system. These results provided a confirmation of the nature of previously proposed stannous-fluoride complexes. They also served to explain why the addition of glycerol, an agent commonly present in dentifrices, protects Sn(II) against oxidation in aqueous solution.

### Silver(I)/Fluoride Ion-Containing Products and Their History: Silver(I)-Diammine Fluoride (SDF)

W. D. Miller identified silver nitrate as the most effective and non-toxic antimicrobial substance effective against oral bacteria in 1890 [14]. Moreover, G.V. Black described in detail his method for arresting tooth decay using silver nitrate in his text entitled “Pathology of The Hard Tissues of The Teeth,” which was published in 1908 [15]. This practice was continued by Percy Howe at the Forsyth Institute [16]. Recent additions to the literature have revisited this approach by combining silver nitrate with fluoride varnish [17].

Silver(I)-diammine fluoride (SDF) was investigated in 1969 by Mizuho Nishino as part of her PhD thesis working in the laboratory of Yamaga [18]. This was a development from an interest in attempting to evolve a combination product, which would capture the antimicrobial properties of silver(I) cation [Ag(I)] and the enamel protectant effects of fluoride [18]. The first commercial product was Saforide, manufactured by Morita in 1970. Saforide is a 38% (w/v) concentration aqueous solution which is, by composition, 25% silver, 8% ammonia, 5% fluoride and 62% water by weight [expressed as (w/w)]. The addition of ammonia was predominantly selected in order to stabilize the product, and to circumvent silver(I) ion precipitation from solution as metallic silver [Ag(0)] or insoluble, black-colored silver(I) oxide (Ag_2_O).

SDF [38% (w/w)] was released into the US market (as Advantage Arrest) by the FDA in 2014 as a desensitization agent. However, clinicians often use this product to arrest active caries in an off-label manner [19]. Given the high fluoride concentration of this product, appropriate consideration must be made with regard to any potential toxic effects exerted by it, most notably when applied in combination with other sources of fluoride. Some authors have promoted an application technique which includes careful drying of the lesion, placement of SDF with a gentle scrubbing motion, followed by covering with a fluoride varnish product to maintain direct contact of the SDF with the treated tooth surface, prevent salivary contamination, and mask the adverse taste sensation known for this product [20].

SDF comprises a two-coordinate complex with the silver(I) ion complexed near-linearly by two ammonia ligands [[Ag(NH_3_)_2_]^+^], apparently with a fluoride counter ion (F^−^). Hence, the molar concentration of SDF in aqueous solution products containing 38% (w/v) of this complex is as high as 2.34 mol./L, with an equivalent molar concentration of fluoride, which translates to a product level of 45,215 to 51,000 ppm F^−^ in clinical sample analyses [21].

In the solid-state, the crystal structure of diamine silver(I) complex with a sulfate counter ion in place of fluoride [[Ag(NH_3_)_2_]^+^)_2_.SO${}_{4}^{2-}$] was re-determined by Zachwieja and Jacobs in 1992 [22]. As expected, these Ag(I) cations were significantly bent, with an N-Ag-N angle of 174.3°, which was ascribable to a degree of oxygen donot atom complexation at its Ag(I) center. Intriguingly, one relevant further crystal structure report found that Ag(I)-coordinated ammonia ligands in the compound [Ag(I)(NH_3_)_2_]F**·**2NH_3_ form strong N–H**···**F hydrogen bonds to adjacent fluoride ions, in addition to weaker N–H**···**N ones to free ammonia ligands [23]. Therefore, one new conjecture from the authors is that in aqueous solution, the [Ag(I)(NH_3_)_2_]^+^ species may effectively aid the delivery of cariostatic fluoride anion to optimal diseased tooth remineralization sites *in vivo*.

**Figure 2:**
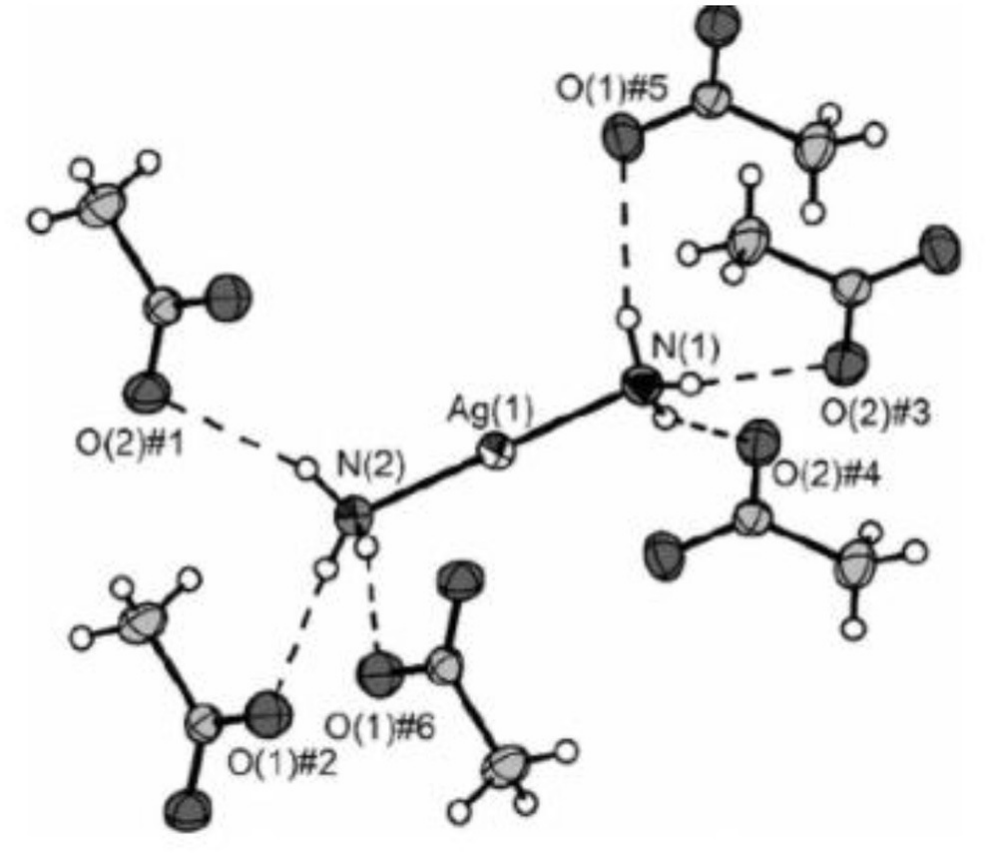
X-ray crystal structure of diammine silver(I) acetate ([Ag(NH_3_)_2_]OAc), which has close to linear two-coordination of silver(I) by two ammonia (ammine) ligands. Reproduced from Ref. [22] with permission.

Similarly, the crystal structure of diamine silver(I) acetate ([Ag(NH_3_)_2_]OAc) demonstrated almost linear [Ag(NH_3_)_2_]^+^ cations (bond angle 176.95°), arranged in a corrugated chain of equidistant silver atoms (Figure 2) [24]. Intriguingly, in this compound, the Ag(I)–Ag(I) distance was found to be within what is described as the “argentophilic” contact range, this indicating that there may be a significant interaction between silver(I) metal ion centers in the solid-state.

Although there are many reports available on the therapeutic properties and efficacies of SDF regarding its value in the treatment of dental caries, such reports are beyond the scope of this review. However, recently Zhao et al. [25] conducted a full and very extensive systematic review of such actions in the oral health research area, and this work primarily identified a grand total of 1,123 publications. Of the 29 peer-reviewed publications selected for further analysis, which explored the influence of SDF on dental hard tissues and cariogenic bacteria, 11 studied the bactericidal properties of SDF, and found that it acted against cariogenic bacteria, predominantly *Streptococcus mutans*, and also suppressed the development of cariogenic tooth biofilms; 20 investigations examined the remineralization of demineralized dentine or enamel by this agent, and found that mineral loss from these sites was retarded following SDF treatment, and that a calcium ion (Ca^2+^) and phosphate-loaded surface was generated on carious lesions which were indeed arrested; finally, 4 reports detailed the protective role of SDF toward dentine collagen, and discovered that it inhibited the actions of collagenases and hence averted collagen degradation. Therefore, the authors of this detailed review concluded that SDF offers very favorable therapeutic effects for each of these three mechanistic considerations.

## Toxicities of Fluorides and Their Excessive Use

### Sodium Fluoride

The harmful effects of fluoride exposure may be placed into two main categories:

Firstly, a massive acute systemic exposure from an industrial accident, and inadvertent ingestions of large quantities of fluoride anion-containing products have been observed [26]. These events can lead to serious injury and death, but thankfully, this type of exposure is very rare. Another type is that chronic exposure to fluoride anion, which can lead to hypomineralized areas of enamel (mottled enamel), which was first recognized as “Colorado brown stain” as noted above [1]. Separate exposures to different types of fluoride compounds are, of course, considered additive, and therefore other fluoride products such as fluoride toothpastes, and the newer product SDF, should be cumulatively considered. Examples of fluoride-containing tap water and oral health products, and their fluoride contents, are provided in Table 1. While the use of fluoridated toothpaste is intended to produce a topical effect, in some situations the toothpaste may be ingested. This is especially a problem for young children who are first learning to brush their teeth. It is therefore universally suggested that just a smear of toothpaste be placed onto the toothbrush of children aged under 6 years, and that adult supervision of tooth-brushing takes place for younger children. There have been cases reported of children eating toothpaste, a situation giving rise to nausea and vomiting.

**Table 1:** Fluoride contents (ppm) of fluoridated tap water and some typical oral health products.

**Source/product**	**[Fluoride] (ppm)**
Water fluoridation	1
Fluoridated toothpaste	1,000–1,500
5% (w/v) sodium fluoride varnish	22,600
38% (w/v) silver diamine fluoride	44,800

Secondly, a mild chronic overexposure to fluoride in children over time may result in some form of fluorosis in developing teeth, and may also cause a transient gastric disturbance [27].

Tooth enamel is porous, and despite having an opacity, is quite transparent; it contains a significant content of protein. Dental fluorosis is one common concern for oral health practitioners, which represents a developmental perturbation of tooth enamel, and which commences during its formation. Unfortunately, fluorosis arises from the excessive systemic exposure of children to fluoride during their first 6 years, a period when permanent teeth crown enamel is generated. Clinical manifestations of fluorosis range from (quantitative) narrow, white horizontally-running lines, more extensive marks, or yellow- to light brown-colored regions of porous enamel, to the qualitatively-visible loss of enamel to varying extents [28, 29]. In order to achieve the optimal therapeutic effects from the use of fluoride toothpastes, it is of much importance for consumers to pay close attention to recommended guidelines available for the employment of products containing such agents. In this manner, the likelihood of fluorosis is diminished, and fluoride's protective effects against the induction and development of dental caries are optimized [1, 30, 31].

The excessive use of fluoride can, however, give rise to acute toxicity, most especially with the ingestion of one or more doses of this agent during a short time duration, which then may give rise to adverse poisoning effects [32]. Primarily, the stomach is affected, and the first symptoms and signs consist of nausea, abdominal pain, bloody vomiting and diarrhea. Subsequently, collapse occurs, together with wetness, paleness, weakness, skin hypothermia, weak heart sounds, shallow breathing, dilated pupils, hypocalcaemia and hyperkalemia, and cyanosis, followed by fatality in some cases within 2–4 h. Further outcomes involve muscle paralysis, together with carpopedal and extremity spasms. From previous investigations of a series of overdose cases, the probable toxic dose (PTD) of fluoride has been stipulated to be 5 mg/kg of body mass (BM) [30]. This PTD value represents the minimal dose that may provoke serious or life-threatening signs and symptoms; such events call for immediate hospitalization and treatment [32]. As an example, a 30 kg pediatric patient would attain the PTD value for fluoride anion if they ingested 150 g (*ca*. 112 mL) of a toothpaste containing 1,000 ppm (mg/kg) of this agent.

A further adverse toxiciological outcome of excessive fluoride ingestion is skeletal fluorosis. Recently, Srivastava and Flora [33] reviewed up-to-date findings focused on skeletal fluorosis and the input of oxidative stress in its progression. This study found that the human consumption of fluoride at concentrations of 1.5 ppm or greater is predominantly responsible for skeletal fluorosis. Indeed, the testing of water supplies from rural areas demonstrated that 80% of villages had water fluoride levels greater than acceptable limits set by the WHO. Indeed, those residing in such areas are afflicted by this condition. Moreover, in many Asian and African regions, endemic fluorosis affects the majority of the populations, i.e., *ca*. 100 million subjects. Skeletal fluorosis represents a slowly progressing disorder, and requires preventative circumvention using mitigation strategies, which indeed have been performed with defluoridation processes globally. Despite being reversible, resolution of such fluoride toxicity and its side-effects is complicated with only very limited treatment options, and these are certainly not affordable in the poverty-stricken rural areas where they are most needed. Since there are no available therapies to effectively combat fluorosis, its direct aversion serves as the best option available. Indeed, the review presented in Ref. [33] discusses the development of relatively simple and economically-viable approaches for fluoride removal from water supplies, in addition to research data available based on strategic therapies for skeletal fluorosis.

Ingested fluoride is predominantly distributed within calcified bone tissues, and is then slowly, albeit cumulatively, recycled during bone remodeling processes. In 1998, Boivin et al. [34] reported a method for the determination of fluoride retention in bone, results from which served as a valuable complement to bone histology for skeletal fluorosis diagnosis and monitoring, and putatively useful for the management of fluoride treatments administered for osteoporosis. Notably, mean fluoride levels were found to be 0.05 and 0.08% (w/w) for two large groups (n >100 each) of untreated osteoporotic patients, treatment duration-dependent 0.24–0.67% (w/w) for 166 osteoporotic patients receiving fluoride therapy, and 0.56–1.33% (w/w) for n = 96 patients with a typical skeletal fluorosis condition. The latter group values were found to be critically dependent on fluorosis etiologies, and associations with the level and duration of fluoride exposure. Overall, throughout prolonged fluoride ingestion episodes, the primary uptake of fluoride by bone is somewhat variable, and is highly linked to remodeling activity. Subsequently, fluoride uptake is enhanced more rapidly, and then attains stability at its maximal level. In order to examine the safety and efficacy of fluoride products, it is necessary to consider the body mass index (BMI) of the patient, together with the precise molecular nature of the fluorido agent applied, and where considered appropriate, a full summation of human exposure to a combination of fluoride-containing products.

### Considerations of the Toxicities of Silver and Silver Ions

Separate considerations should be made for the potential toxic effects of silver and its Ag(I) cation. Essentially, silver(I) ions are very toxic to bacteria *via* diffusion through cell walls and cell membranes, and are either specifically or non-specifically complexed by selected amino acid residues present in intracellular or extracellular proteins, and/or by the purine and pyrimidine base moiety, or phosphate ligands, in DNA. These processes denature these biomolecular substrates, and also cause bacterial metabolism to be disrupted [35]. Ag(I) ion has quite a strong affinity for sulfur donor atoms present in the thiol function of the amino acid L-cysteine [36], and such interactions represent a key mechanistic action regarding the bactericidal properties of this metal ion.

Since humans are entirely covered by some form of epithelium (dead cells), silver ions bind to epithelial cells and do not gain access to the intracellular spaces, a process rendering them non-toxic. However, in cases of extremely high exposure to silver(I) ions, a condition known as argyria may develop. In these rare cases of silver exposure, silver is deposited throughout organ systems, and the skin will exhibit a pale blue-gray coloration [37], the case reported in this reference involving the accidental ingestion of colloidal silver. Moreover, accidental skin exposure to silver nitrate and SDF generates a temporary superficial stain similar to a “henna tattoo.” Fortunately, this stain, which appears to largely consist of metallic silver [Ag(0)], eventually disappears with normal skin cell exfoliation. Recent studies, however. have explored the possible toxic effects of silver(I) ion exposure from SDF [38]. The silver content of small amounts of SDF used to treat tooth decay results in a very low exposure, and therefore systemic silver toxicity should not represent a major concern to clinicians. However, most SDF products available are caustic (with pH values ranging from 10 to 13), and therefore could cause a chemical “burn” to the epithelial lining or conjunctiva of the eye, if indeed an inadvertent spillage were to occur. Both operators and patients should, of course, be wearing eye protection during treatment episodes.

## Estimations of Fluoride Exposures and Toxicities For Differential Fluoride-Containing Oral Healthcare Products: Quantitative Problems Experienced by Dental Clinicians

**Figure 3:**
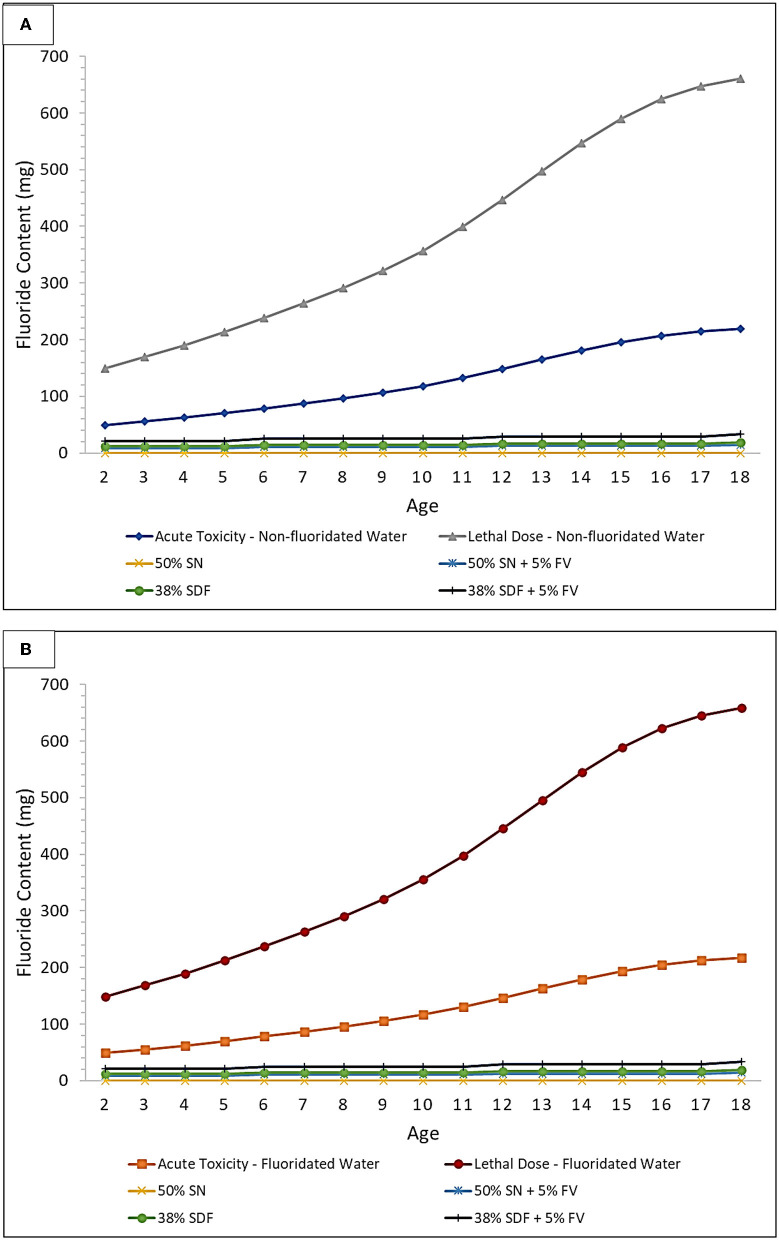
Acute toxicity and CLD thresholds of fluoride for silver nitrate (SN) and SDF both with and without a sodium fluoride-containing varnish (FV) in **(A)** non-fluoridated and **(B)** fluoridated water environments. Treatment comparison against the fluoride Acute Toxicity and CLD Thresholds (including considerations for the ingestion of fluoridated water, or not, and also toothpaste up to the age of 6 years) using 10 μl of 50% (w/v) SN, or 10 μl of 38% (w/v) SDF, and both with and without the application of 20 μl of a 5% (w/v) FV. These plots were derived using the assumption that every tooth is treated for each age group considered, and all ages are within the 3% weight group, to further demonstrate the safety margin in a worst-case-scenario.

**Figure 4:**
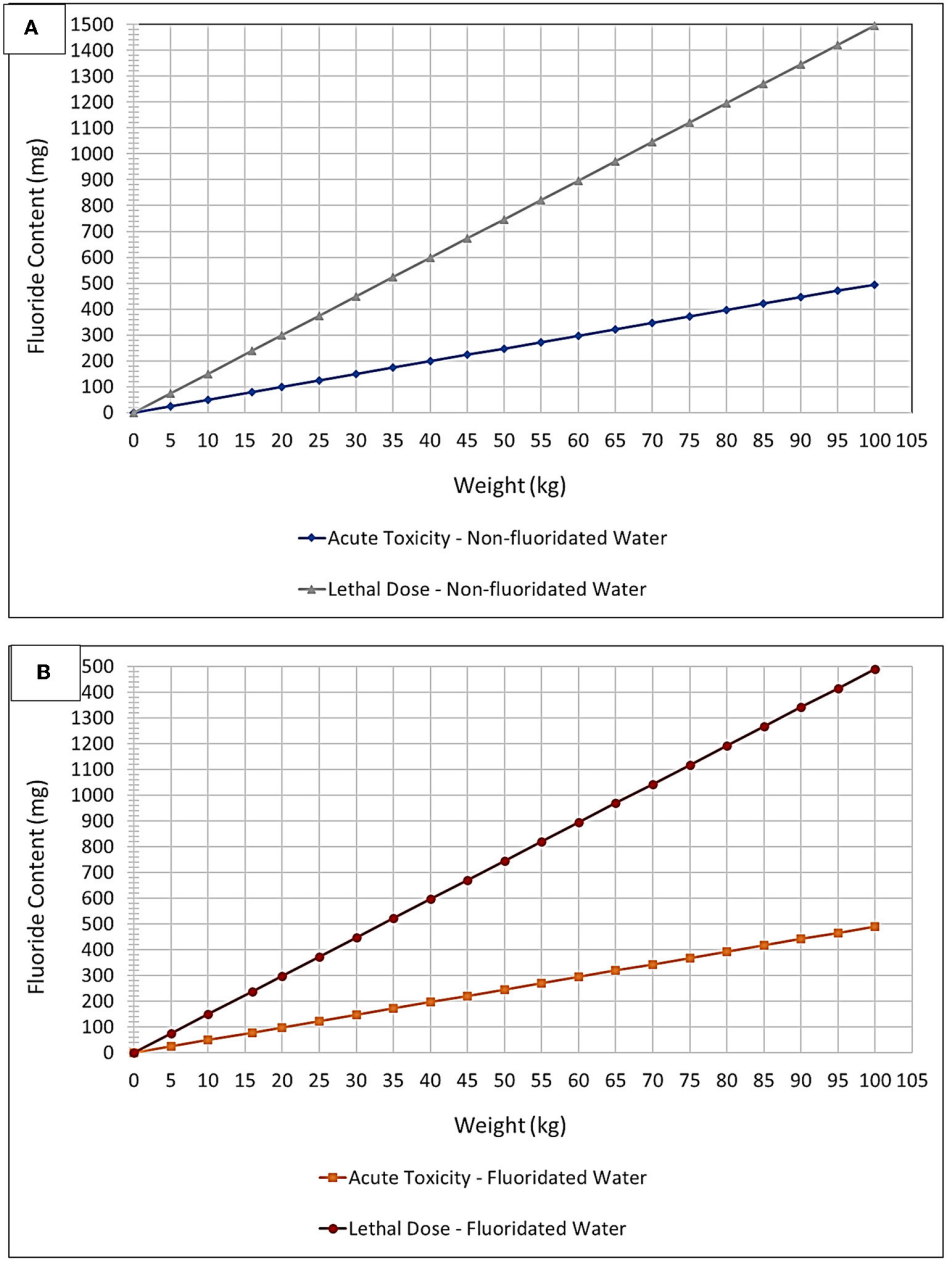
“Plug and play” plot option – fluoride toxicity. Acute Fluoride Toxicity and CLD Thresholds expressed as a plot of mg of potential fluoride ingested vs. child body weight (kg) for **(A)** non-fluoridated and **(B)** fluoridated water environments. These thresholds were adjusted to account for fluoridated and non-fluoridated water environments, in addition to the ingestion of toothpaste up to an age of 6 years based on a mean weight of 16 kg (lowest 3% body weight average).

The potential for various human exposures to fluoride from differential oral healthcare product sources presents a highly challenging situation for clinicians who are attempting to optimize the benefits of fluoride against its potential negative side-effects, such as gastritis and enamel mottling. In addition, the various common units available for expressing or displaying fluoride concentrations may lead to some confusion amongst dental practitioners, for example through the use of % compositions [both (w/w) and (w/v)], parts-per-million (ppm) and moles per liter (mol./L, i.e., molarity) concentration units, etc. Therefore, the diagrams and Tables provided below (Figures 3, 4, and Tables 2, 3) aim to provide a simple and rapid means of estimating the exposure of patients based on body mass index (BMI) and their various fluoride exposure patterns. The overall goal of this strategy is to easily inform both the clinician and patient of the likelihood of any potential toxic outcomes for the use of fluoride compounds during such dental treatments.

**Table 2:** Fluoride content (mg) based on number of teeth treated and protocol used (SN or SDF, both with and without FV).

**Teeth**	**50% SN**	**50% SN + 5% FV**	**38% SDF**	**38% SDF + 5% FV**
1	0.00	0.45	0.59	1.04
2	0.00	0.90	1.18	2.08
3	0.00	1.36	1.77	3.13
4	0.00	1.81	2.36	4.17
5	0.00	2.26	2.95	5.21
6	0.00	2.71	3.54	6.25
7	0.00	3.16	4.13	7.29
8	0.00	3.62	4.72	8.34
9	0.00	4.07	5.31	9.38
10	0.00	4.52	5.90	10.42
11	0.00	4.97	6.49	11.46
12	0.00	5.42	7.08	12.50
13	0.00	5.88	7.67	13.55
14	0.00	6.33	8.26	14.59
15	0.00	6.78	8.85	15.63
16	0.00	7.23	9.44	16.67
17	0.00	7.68	10.03	17.71
18	0.00	8.14	10.62	18.76
19	0.00	8.59	11.21	19.80
20	0.00	9.04	11.80	20.84
21	0.00	9.49	12.39	21.88
22	0.00	9.94	12.98	22.92
23	0.00	10.40	13.57	23.97
24	0.00	10.85	14.16	25.01
25	0.00	11.30	14.75	26.05
26	0.00	11.75	15.34	27.09
27	0.00	12.20	15.93	28.13
28	0.00	12.66	16.52	29.18
29	0.00	13.11	17.11	30.22
30	0.00	13.56	17.70	31.26
31	0.00	14.01	18.29	32.30
32	0.00	14.46	18.88	33.34

*From this table, clinicians can readily determine how much fluoride (in mg) is potentially available for ingestion, depending on the number of teeth being treated and which protocol is being employed. Once the quantity of fluoride in mg has been determined, it can be used in the “plug-and-play” plot option (Figure 4), along with the child's weight, to determine if the amount of fluoride to be administered is above or below the Acute Toxicity and CLD Thresholds. This table was designed assuming an application volume of 10 μL of 50% (w/v) SN, 10 μl of 38% (w/v) SDF, and/or 20 μl of 5% (w/v) FV. The amount of fluoride in 38% (w/v) SDF was calculated using the higher limit of the fluoride content range present in different SDF products [5.0–5.9% (w/v)] to again demonstrate the safety margin in a “worst-case-scenario.” The total amounts of fluoride present in 3.00 and 8.00 mL volume bottles of a well-known SDF product containing 38% (w/v) of this agent (Advantage Arrest) is 177 and 472 mg, respectively*.

**Table 3:** Amount of fluoride from toothpaste ingested by children based on age.

	**Fluoride from ingested toothpaste—(mg/day)**	
	**Pea-sized**	**Strip**
<6 years old	0.15	0.6
>6 years old	0	0

*This table assumes children brush twice daily, children under 6 years old use a pea-sized amount of toothpaste (0.075 mg fluoride), and children over 6 years old use a strip-sized amount of toothpaste (0.3 mg fluoride). Additionally, this table also assumes that children over 6 years old generally do not ingest toothpaste when brushing their teeth, whereas those under 6 years old do so. Figures 3, 4, along with Table 2, incorporate this amount of ingested fluoride [39]*.

### Working Relationship of Fluoride Exposure to Toxicity

In Figure 3, the acute fluoride toxicity and Certainly Lethal Dose (CLD) threshold curves are demonstrated for the therapeutic application of silver nitrate and SDF treatments, both with and without the co-application of a fluoride varnish; both fluoridated and non-fluoridated water environments are considered. The lethal dose value employed was that available in Ref. [40], specifically 15 mg/kg. Childrens' weights at each age were selected to be the lowest 3% for each age group in order to provide a “worst-case-scenario” to provide an improved portrayal of the safety margin. This was then used to determine how much fluoride would have to be ingested to meet the 5 mg/kg body weight PTD acute fluoride toxicity and CLD thresholds. In addition, since different age groups have differing average numbers of teeth, this was accounted for when determining how many mg of fluoride is available for ingestion during treatments. This plot assumes that every tooth is treated using a 10 μl “drop” of 38% (w/v) SDF [41], or alternatively 50% (w/v) silver(I) nitrate (SN) and a 20 μL volume of 5% (w/v) sodium fluoride varnish (FV) [42], since these are approximately the volumes that are employed in the field. It should also be borne in mind that 38% (w/v) SDF has a fluoride content ranging from 5.0 to 5.9%, and this value is dependent on the brands and batches utilized. The calculations for Figure 3 were performed using a 5.9% fluoride content value in order to optimize a demonstration of the safety margin. Notably, however, treatment of every tooth using any of the protocols shown places all patients well within the safety margin. This safety margin obviously increases as children become older with greater body masses.

Figure 4 and Table 2, however, offer a “plug-and-play” option for practicing clinicians who wish to know exactly how close to the acute fluoride toxicity threshold a child patient will be when undergoing such treatment, and this is critically dependent on the protocol used, the number of teeth treated, and the child's body weight. A small child with a large number of teeth, for example, may have to have only half of their teeth treated to ensure that their exposure to fluoride isn't too high during that treatment session. Taken together, they offer a tool for clinicians to facilitate the proper instigation of a safe treatment plan featuring these treatment protocols. Moreover, this plot accounts for both fluoridated and non-fluoridated water environments. By viewing the amount of fluoride received during a certain treatment protocol (derived from Table 2) as a function of the patients' body mass, a clinician can readily determine its safety margin expressed relative to fluoride Acute Toxicity and CLD Thresholds.

Table 3 shows exactly how much fluoride can be ingested by children depending on how old they are and what amount of toothpaste they are using during toothbrushing episodes. However, it should also be noted that children under the age of 6 years generally ingest approximately one-half of the quantity of toothpaste used, whereas children older than this generally do not swallow significant amounts of their toothpaste products [39].

Using Table 2, a clinician can determine how many mg of fluoride is potentially ingestible based on the protocol used and the number of teeth being treated.

## Discussion

The relationship between the dental profession and fluoride has been long and complex. It began over 100 years ago when it was first speculated that an unknown substance in the water of communities in Colorado, USA was causing enamel staining (mottling). Subsequently, a correlation was made between mottling incidence and reduced caries rates, and careful analysis demonstrated that fluoride anion was involved in both processes. A concerted effort then proceeded to determine how to balance the protective effects of fluoride against its potential negative outcomes. Particularly notable is the addition of the novel “metallodrug”-type agent SDF to the World Health Authority's (WHO's) list of essential medicines targeted on the prevention and treatment of tooth decay [43].

Presently, we find ourselves in a situation where fluoride has been added to community water supplies, toothpaste and other therapeutics. During this period of time, no standard measurement units have been used when demonstrating the concentration of fluoride in these products. Sometimes, a percentage of net weight or volume is used, and sometimes a parts-per-million (ppm) metric has been employed. This often leads to the confusion of clinicians and the general public when they try to judge the safety and efficacy of fluoride-containing products and materials which they use during their daily working lives. There are even examples where certain groups have based their political views on fluoride supplementation. A full review of no fewer than 87 cases of toxic exposure to high levels of fluoride by children have been reported in the literature [44]. In this study, 84 out of 87 cases featured the accidental ingestion of fluoride-containing oral healthcare products, i.e., drops, rinses and tablets, etc., at home by children aged up to 6 years old. However, two children aged 8 and 9 years old were reported as becoming symptomatic following treatment with fluoride by a dental clinician. The single remaining case involved the fatal ingestion of an unknown quantity of an insecticide containing sodium fluoride by a child aged 13 months (a substantial decline in blood serum calcium concentration was observed post-mortem). Approximately one-third of the total of 87 child cases displayed gastrointestinal (*n* = 25) and drowsiness (*n* = 1) symptoms, the former including nausea, vomiting, abdominal pain and diarrhea; 3 of these only became symptomatic at a time-point >1 h subsequent to ingestion.

A central theme of this report is to enable clinicians to identify the margin of safety that a patient may experience based on body mass index and exposure to various fluoride-containing products. Indeed, the included graphical Figure diagrams and Tables are valuable for the rapid estimation and recognition of such margins in order to provide solutions in “on-the-spot” clinical situations at points of patient contact. Hence, this information will serve to assist clinicians with the taking of informed decisions regarding the application or prescription of fluoride-containing products. Another major objective of this study was to assist the public in the evaluation of this controversial subject through common dialogue with their dental clinicians, a process which will hopefully lead to a more complete general understanding of the subject matter.

## Potential Limitations of the Study

Firstly, the local water fluoridation level is a factor which will always depend on the water intake of an individual, and which may vary based on environmental temperature, and differential between-subject water consumption levels ascribable to exercise and other requirements, etc. Notably, previous scientific reports based on this subject have often utilized group estimates rather than individual quantitative data available.

Secondly, the unintended systemic exposure to fluoride in toothpaste covers a wide range of situations. For example, are parents sufficiently educated regarding the amount of toothpaste to place on the brush, as in the well-known “smear” approach, and do children purposely swallow toothpaste because they are fond of the taste, or other explanations?

Thirdly, the fluoride content involved in the applications of SDF to caries lesions should be considered, the treatment intent being their topical administration to these locations. However, there may be some inadvertent systemic absorption of fluoride; indeed, the transient systemic presence of fluoride following SDF application has been reviewed in Ref. [41].

Finally, possible variations in exposure level should not deflect from the importance and usefulness of making realistic predictions of acceptable and safe values for this critically important parameter. However, the wide safety margins demonstrated in previous studies should provide reassurance to clinicians who wish to embrace any newly-developed fluoride therapies.

## Conclusions

In this manuscript, we present a simple graphical plot/tabular tool which allows clinicians to gauge the overall exposure of their patients to fluoride-containing products with respect to both acute and chronic toxicities. This tool will undoubtedly also assist clinicians who wish to discuss these issues with adult patients, and parents of child patients, about fluoride anion, fluoride adducts, and their potential, albeit very unlikely, toxic effects.

After over 100 years of discovery, and the clinical application of fluoride products into clinical practice, this field continues to evolve with new knowledge and therapeutics, most especially with the design of novel fluoride-containing and -delivery compounds such as cariostatic SDF. A periodic review of this history, and current tools for clinical practice, are indicated here for the benefit of both patients and clinicians. Relevant information concerning the molecular structures, solution status and potential mechanisms of action of all fluoride derivatives employed in oral health, such as MFP, stannous fluoride and SDF is also presented, as is information on their possible, albeit very unlikely, adverse health effects. Fluoride is now commonly present in diverse sources such as community water systems, toothpastes and topical products, as well as new therapeutics, and therefore the development and use of a simple graphical tool to estimate possible fluoride-induced toxicities serves as a major benefit for practicing dental clinicians, together with oral healthcare workers in general.

## Data Availability Statement

The raw data supporting the conclusions of this article will be made available by the authors, without undue reservation.

## Author Contributions

SD and MD: conceptualization, resources, writing—original draft preparation, and project administration. SD, MD, and MG: methodology, software, validation, investigation, data curation, writing—review and editing, and writing—final draft preparation. MD and MG: formal analysis and visualization. All authors have read and agreed to the published version of the manuscript.

## Conflict of Interest

Authors SD and MD were employed by Shoreview Dental LLC, Oral Health Outreach LLC, and NoDK LLC. The remaining author declares that the research was conducted in the absence of any commercial or financial relationships that could be construed as a potential conflict of interest.

## Publisher's Note

All claims expressed in this article are solely those of the authors and do not necessarily represent those of their affiliated organizations, or those of the publisher, the editors and the reviewers. Any product that may be evaluated in this article, or claim that may be made by its manufacturer, is not guaranteed or endorsed by the publisher.
